# 

**Published:** 1897-09

**Authors:** 


*The following donations have been received since the publication of the list in June:*

*August 31st, 1897.*

| | |
|---|---|
| Columbian University (1) ... ... ... ... ... ... | 1 volume. |
| Detroit Public Library (2) ... ... ... ... ... ... | 18 volumes. |
| L. M. Griffiths (3) ... ... ... ... ... ... ... ... | 5 ,, |
| Pathological Society of London (4) ... ... ... | 1 volume. |
| Royal Medical and Chirurgical Society of London (5) ... ... ... ... ... ... ... ... ... | 8 volumes. |
| Smithsonian Institution (6) ... ... ... ... ... | 20 ,, |

Reprints have been received from the Smithsonian Institution, and unbound periodicals from the Detroit Public Library. Mr. L. M. Griffiths, Dr. E. Markham Skerritt, and Mrs. Greig Smith have each presented a framed picture.

### TWENTY-FIFTH LIST OF BOOKS.

The titles of books mentioned in previous lists are not repeated.

The figures in brackets refer to the figures after the names of the donors, and show by whom the volumes were presented. The books to which no such figures are attached have either been bought from the Library Fund or received through the *Journal*.

| | | | |
|---|---|---|---|
| **Adams, W.** | *Subcutaneous Surgery* | (6) | 1877 |
| **Allbutt, T. C.** [Ed.] | *A System of Medicine.* Vol. III. | | 1897 |
| **Allen, H.** | *A Clinical Study of the Skull* | (6) | 1890 |
| **Ball, J. S.** | *Diseases of the Nose and Pharynx* | 3rd Ed. | 1897 |
| **Bell, B.** | *A System of Surgery.* 7th Ed., Vols. II., IV.—VII. | (3) | 1801 |
| **Binz, C.** | *Lectures on Pharmacology.* Vol. II. (Tr. from 2nd German Edition by P. W. Latham) | | 1897 |
| **Bolton, H. C.** | *A Select Bibliography of Chemistry*, 1492–1892 | (6) | 1893 |
| **Bramwell, B.** | *Diseases of the Spinal Cord* | 3rd Ed. | 1895 |
| ,, | *Atlas of Clinical Medicine.* Vol. III. | | 1896 |
| **Brown-Séquard, C.E.** | *Dual Character of the Brain* | (6) | 1877 |
| **Brunton, T. L.** | *The Action of Medicines* | | 1897 |
| **Clark, A. C.** | *Mental Diseases* | | 1897 |
| **Da Costa, J. M.** | *On Strain and Over-Action of the Heart* | (6) | 1874 |
| **Dean, J.** | *The Gray Substance of the Medulla Oblongata and Trapezium* | (6) | 1869 |
| **Dowse, T. S.** | *The Pocket Therapist* | | 1897 |
| **Erskine, J.** | *A Plea for a State Medical Service* | | 1897 |
| **Giles, J. B. Sutton and A. E.** | *Diseases of Women* | | 1897 |
| **Haig, A.** | *Uric Acid in Causation of Disease* | 3rd Ed. | 1896 |
| **Harbitz, F.** | *Om Endokardit* | | 1897 |
| **Hedley, W. S.** | *Practical Muscle Testing* | | 1897 |
| **Hewer, Mrs. Langton.** | *Our Baby* | 5th Ed. | 1897 |
| **Holthouse, E.** | *Convergent Strabismus* | | 1897 |
| *Isle of Man, The—Official Guide* | | | [N.D.] |
| **Jewett, C. C.** | *Notices of Public Libraries in the United States of America* | (6) | 1851 |
| ,, | *On the Construction of Catalogues of Libraries* | (6) 2nd Ed. | 1853 |
| **Kanthack, H. D. Rolleston and A. A.** | *Practical Morbid Anatomy* | | 1894 |
| **Keen, W. W.** | *Surgical Complications and Sequels of Fevers* | (6) | 1877 |
| **Lewis, P. G.** | *Spinal Curvatures* | | 1897 |
| **McAdie, A.** | *Equipment and Work of an Aero-Physical Observatory* | (6) | 1897 |
| **Mitchell and G. R. Morehouse, S. W.** | *Anatomy and Physiology of Respiration in the Chelonia* | (6) | 1863 |
| **Moor, T. H. Pearmain and C. G.** | *The Analysis of Food and Drugs.* Part I. | | 1897 |
| **Morehouse, S. W. Mitchell and G. R.** | *Anatomy and Physiology of Respiration in the Chelonia* | (6) | 1863 |

**Morgan, L. H.** ... *Circular in Reference to the Degrees of Relationship among Different Nations* ... ... ... ... (6) 1860
**Moure, E. J.** ... *De l'Ouverture large de la Caisse et de ses Annexes* ... 1897
**Muir and J. Ritchie, R.** *Manual of Bacteriology* ... ... ... ... ... ... ... 1897
**Owen, E.** ... ... *The Surgical Diseases of Children* ... ... ... 3rd Ed. 1897
**Pearmain and C. G. Moor, T. H.** *The Analysis of Food and Drugs.* Part I. 1897
**Ranney, A. L.** ... *Eye-Strain in Health and Disease* ... ... ... ... ... 1897
**Reynolds, S. H.** ... *The Vertebrate Skeleton* ... ... ... ... ... ... ... 1897
**Rhees, W. J.** ... *Catalogue and Index of the Publications of the Smithsonian Institution,* (1846–1882) ... ... ... (6) 1882
**Ritchie, R. Muir and J.** *Manual of Bacteriology* ... ... ... ... ... ... 1897
**Robson, A. W. M.** *Diseases of the Gall-Bladder and Bile-Ducts* ... ... ... 1897
**Rolleston and A. A. Kanthack, H. D.** *Practical Morbid Anatomy* ... ... 1894
*Royal Medical and Chirurgical Society, Catalogue of the Library of the.* Supplements I.—VIII. ... ... ... (5) 1880–96
**Sergi, G.** ... ... *The Varieties of the Human Species* ... ... ... (6) 1894
**Shakespeare, E. O.** *Reparatory Inflammation in Arteries* ... ... ... (6) 1879
*Sion College Library, London, Order of Classification of* ... ... ... 2nd Ed. 1889
**Smith, W. R.** ... *The Laboratory Text-Book of Public Health* ... ... 1896
*Smithsonian Institution, Catalogue of Publications of Societies and of Periodical Works belonging to the* ... ... ... ... ... (6) 1866
**Sutton and A. E. Giles, J. B.** *Diseases of Women* ... ... ... ... ... ... 1897
**Turner, D.** ... ... *Practical Medical Electricity* ... ... ... ... 2nd Ed. 1897
**Walsh, D.** ... ... *Excretory Irritation* ... ... ... ... ... ... ... ... ... 1897
**Waring, Jr., G. E.** *Sanitary Drainage of Washington City* ... ... (6) 1880
**Wilson, E. M.** ... *Notes on Malaria* ... ... ... ... ... ... ... ... ... 1897
**Wood, H. C.** ... ... *A Study of Fever* ... ... ... ... ... ... ... ... (6) 1875
,, ... ... *Fever: a Study in Morbid and Normal Physiology* (6) 1880
**Woodward, J. J.** ... *On Cancerous Tumors* ... ... ... ... ... ... (6) 1873

---

### TRANSACTIONS, REPORTS, JOURNALS, &c.

American Journal of Obstetrics, The ... Vols. (2) XIX., 1886; XXXV. 1897
American Journal of the Medical Sciences, The... ... ... Vol. CXIII. 1897
American Lancet, The (2) Vols. XIII., XIV., 1889–90, XVII., XVIII. 1893–94
American Medico-Surgical Bulletin ... ... (2) Vols. VII., VIII. 1894–95
Boston Medical and Surgical Journal, The (2) Vols. CXVI., 1887, CXX. 1889
Bristol Health Report, 1896 ... ... ... ... ... ... ... ... ... ... 1897
British Gynæcological Journal, The ... ... ... ... ... Vol. XII. 1896
British Medical Journal, The ... ... ... ... ... ... ... Vol. I. for 1897
China Medical Missionary Journal, The ... ... ... ... ... Vol. X. 1896
Cincinnati Lancet-Clinic, The... ... ... ... ... ... Vol. XXXVIII. 1897
Columbian University, Catalogue of the ... ... ... ... ... (1) 1897
Dictionary of Bathing Places, Bradshaw's... ... ... ... ... ... ... 1897
Dublin Journal of Medical Science, The ... ... ... ... Vol. CIII. 1897
Edinburgh Medical Journal, The ... ... ... ... ... ... N.S., Vol. I. 1897
Epitome, The ... ... ... ... ... ... ... ... (2) Vols. VIII., 1887, X. 1889
Grant College Medical Society, Transactions of the, 1896 ... ... ... 1897
Hospital and Charities Annual, Burdett's ... ... ... ... ... ... ... 1897

Hospitals—

Cincinnati Hospital, Thirty-sixth Annual Report of the, for 1896 1897

King's College Hospital Reports ... ... ... ... ... Vol. III. 1897

Saint Bartholomew's Hospital Reports ... ... ... Vol. XXXII. 1897

Journal of the American Medical Association, The ... Vol. XXVIII. 1897

Lancet, The... ... ... ... ... ... ... ... ... ... ... ... Vol. I. for 1897

Laryngoscope, The ... ... ... ... ... ... ... ... ... ... Vol. II. 1897

Medical News, The ... ... ... ... ... ... ... ... ... Vol. LXX. 1897

Medical Press and Circular, The ... ... ... ... ... ... [Vol. CXIV.] 1897

Medical Record ... ... ... ... ... ... ... ... ... ... ... ... Vol. LI. 1897

Medical World, The... ... ... ... (2) Vols. VII., 1889, IX., X. 1891-92

Montreal Medical Journal, The ... ... ... ... ... Vol. XXV. [1896-97]

New York Academy of Medicine, Transactions of the 2nd Ser., Vol. XI. 1895

New York Medical Journal, The ... ... ... ... ... ... Vol. LXV. 1897

New York, Transactions of the Medical Society of the State of ... ... 1897

Obstetrical Society of London, Transactions of the ... Vol. XXXVIII. 1897

Pathological Transactions for Vols. XVI. to XXV., 1865–74, Index to (4) 1875

Physician and Surgeon, The (2) Vols. XIII., XIV., 1891–92; XVII., XVIII. 1895-96

Practitioner, The ... ... ... ... ... ... ... ... ... ... Vol. LVIII. 1897

Progrès Médical, Le ... ... ... ... ... ... ... 3e Sér., Tome V. 1897

Quarterly Epitome of American Practical Medicine and Surgery (2) [Vols. V., VI.] 1884-85

Retrospect of Medicine, Braithwaite's ... ... ... ... ... Vol. CXV. 1897

Revue hebdomadaire de Laryngologie, d'Otologie et de Rhinologie Tome XVII., Vol. I. 1897

Sanitary Record, The ... ... ... ... ... ... ... ... Vol. XIX., 1896-97

Smithsonian Institution, Annual Report of the, 1895 ... ... ... ... 1896

---

### Local Medical Notes.

### BRISTOL.

Health Report.—We give below a *précis* of the report of the Medical Officer of Health, and can congratulate the city on the healthy condition of the inhabitants during the year 1896.

The population of the city is now calculated to be 230,623; but, as pointed out in previous reports, this does not really represent the figures for Bristol: the present year will show a great increase, consequent on the enlargement of the boundaries. Under the head of sewerage, some remarks on the state of the Avon do not come amiss. It appears that, owing to complaints made in the summer, a sub-committee enquired exhaustively into the question of the disposal of the sewage; but we are not told what were the conclusions arrived at, or what, if any, scheme was proposed for the allaying of the nuisance, which, if not detrimental to health, is offensive to the senses. There is no doubt that, with a population increasing at the present rate, some means must be found for disposing of the sewage other than the present; but if the inhabitants require it, they must be prepared to meet the cost, which cannot be a small one.

The two new *isolation hospitals* are making progress, and when completed will raise the available accommodation to 208 beds. Dr. Davies points out that the city will be adequately provided when it has 230 beds, but with the increased size of the city the number required will be considerably more.

The vital statistics show a decrease in the *birth-rate*, but we hope with the increased *marriage-rate* of 1896 this may be remedied before the next report is issued. The morality of Bristol appears to be improving, to judge from the decrease in illegitimate births. The *death-rate* has decreased from 18.08 in 1895 to 16.84 in 1896, while the *infant mortality-rate*, always a heavy item, has decreased from 141.19 to 135.9. These facts are very satisfactory. Last year we congratulated 302 persons on being alive when they should have been dead; this year we have to do the same to 455 persons, who, according to the averages, should no longer be with us. The increased number of deaths from cancer and diseases of the circulatory system is maintained; but the figures are more satisfactory than those of last year, while the decrease in diseases of the respiratory system is more favourable. The increase in deaths from diphtheria, unfortunately, is maintained, but the statistics, on the whole, show that a very considerable saving of life has been effected when compared with the returns of 1895 or of the preceding decennium.

The *vaccination* statistics are, as usual, for the year previous to the report. In 1895 the percentage of successful vaccinations in the Bristol Union was 73.06 (an increase of 1.57), which compares favourably with the percentage for England and Wales (72.3). A new feature in the Report is a report from the Stapleton Workhouse, which during the year was furnished with a resident medical officer. From this may be gathered an instructive lesson. A case of small-pox occurred in a ward containing 90 persons, and the medical officer vaccinated the remaining 89, except one "old man" of 58 who refused, and took the disease in the confluent form. In all, the medical officer vaccinated 496 persons with 4 failures and 2 unknown results, so that the disease was confined to 4 persons. In the Barton Regis Union the percentage of successful vaccinations was 69.6,—an increase on 1894 when it was 68.6, but not so high as 1893 when it was 72.0.

The fatal cases of *diarrhœa* were 106, as against 143 in 1895.

There were 38 fatal cases of *diphtheria* against 34 in 1895; but, the case-mortality shows a decrease.

| | | Cases. | Deaths. | Case-mortality. |
|---|---|---|---|---|
| 1896 | ... | 258 | 38 | 14.7. |
| 1895 | ... | 165 | 34 | 20.6. |
| 1894 | ... | 118 | 50 | 39.0. |

Does this mean a less virulent disease or the result of antitoxin?

We notice that there are still a considerable number of cases notified as diphtheria or membranous croup in which the diagnosis is not confirmed by bacteriological examination. In the laboratory 160 notified cases and 40 unnotified ones were examined with a positive result in 83 cases, a very low percentage. Some curious instances are given in which the examination failed to show the bacillus in what appeared to be undoubted cases from the clinical features.

For fourteen months, from July 9, 1894, the city was free from *small-pox*, when on Sept. 16, 1895, a case appeared at the Long Ashton

Workhouse. Three subsequently were found at Barton Regis Workhouse and others in the Stapleton district, while two more occurred in December. During the year 1896 36 cases occurred in the city, 26 of which were clearly traced to infection from Gloucester. To show how energetic are the means taken to combat the spread of the disease, it is only necessary to mention that over 3,000 visits were paid to the common lodging-houses during the first six months of the year. A short note is added on the Gloucester epidemic, showing that of 789 unvaccinated persons 313 died, or 1 in every 3 attacked; while of the vaccinated 1,208 only 113, or less than 1 in 10.

The number of cases of *scarlet fever* increased from 562 in 1895 to 1,352 in 1896; and it is hoped that with the further hospital accommodation this number will be reduced in future. *Enteric fever* claimed more cases, but the mortality was lower; while *typhus* was again conpicuous by its absence. The deaths from *measles* were 143, an increase on 1895; from *whooping-cough*, 64; and from *phthisis*, 320.

Comparing Bristol with other large towns, it must be admitted that the city comes out very well, but for actual figures we must refer our readers to the Report.

Part II. consists of the report of the Chief Inspector of Nuisances, admirable as evidence of the work done, and showing how the old abominations of our ancestors are being cleared away by the present generation.

The meteorological report by Mr. Rintoul as usual concludes the Report. The chief peculiarities of the year were the warm months of April, May, June, and July, and the slightly deficient rainfall. The hottest day of the year was June 15, when the thermometer reached 84.2°.

In the *Port Sanitary District Report* we learn how energetically means are taken to prevent the introduction of disease, and particularly cholera, by the ships visiting our ports. We regret to see that the deputation to the Chancellor of the Exchequer failed to obtain any assistance from the Imperial funds towards the expenses incurred under the Special Cholera Regulations. It certainly does not appear to be just that the citizens of the ports of Great Britain should bear the whole expense of what is done for the protection of the kingdom generally, and we hope that the permanent officials of the Local Government Board will on a future occasion advise the Chancellor differently. Though there was no cholera scare in 1896 the precautions have never been relaxed, and in all ships bound from certain scheduled ports the authorities have been on the look-out for cholera, yellow fever, and plague. To give some idea of the amount of work done by the inspectors, we may say that 1,675 ships were inspected in detail, of which 292 had one or more sanitary defects.

The steam launch *Luath* seems to be often in trouble.

University College.—The following students have recently passed examinations:

**University of London.**—*Intermediate Examination*—R. G. Johnson; *Physiology only*—P. G. Stock.

**Examining Board in England by the Royal Colleges of Physicians and Surgeons.**—*First Examination. Part I. Chemistry and Physics*—W. B. Barnes, J. A. Bartlett; *Part II. Practical Pharmacy*—E. V. Foss, R. H. N. Rutherford; *Part III. Elementary Biology*—K. H. Beverley, T. S. Davies, E. J. Dermott, F. W. Morgan. *Second Examination. Anatomy and*

*Physiology*—S. J. Willcox; *Anatomy only*—J. E. Jones, S. M. Dowling.

**Army Medical Staff.**—W. P. Gwynn.

At Netley, in July, Mr. H. L. W. Norrington's combined marks secured him the ninth place on the list.

Bristol Royal Infirmary.—G. Munro Smith, M.R.C.S. Eng., Assistant-Surgeon, has been appointed Surgeon. T. Carwardine, M.S., M.B. London, F.R.C.S. Eng., has been appointed Assistant-Surgeon. J. O. Symes, M.D. Lond., D.P.H., has been appointed Honorary Bacteriologist. Harold F. Mole, F.R.C.S. Eng., House-Physician, has been appointed House-Surgeon. E. H. E. Stack, M.B., B.C. Cantab., F.R.C.S. Eng., has been appointed House-Physician.

Greig Smith Memorial.—It was only right and proper that some sort of memorial should be made of the eminent surgeon whose early decease we had to record in our last number, but it has been found a more difficult thing to decide what form the memorial should take than to collect the necessary funds. The idea of doing something originated, as perhaps is not unnatural, among the friends who were associated with him at the Royal Infirmary, and at their instigation a Committee was formed to take the matter in hand. The circular sent out early in August has resulted in a not inconsiderable sum being promised. After the death of Greig Smith a mask was taken of his features, and from this, with other aids, it is hoped to produce a satisfactory bust, from which a bronze can be cast and placed perhaps in some well-known building in the city. With the balance of the money it is proposed to renovate the operating theatre at the Royal Infirmary, and so make it more in accordance with the present day ideas of what such a room should be. The Committee inclined to this proposal because it was well known that Greig Smith had the matter very much at heart. Possibly some memorial such as a medallion may be placed in the theatre.

### SCRAPS

### PUBLICATIONS RECEIVED.

From BAILLIÈRE, TINDALL AND COX, London:

*Mental Diseases.* By A. CAMPBELL CLARK, M.D. 1897.
*Excretory Irritation.* By DAVID WALSH, M.D. 1897.
*The Analysis of Food and Drugs.* Part I. *Milk and Milk Products.* By T. H. PEARMAIN and C. G. MOOR, M.A. 1897.
*Diseases of the Nose and Pharynx.* By JAMES B. BALL, M.D. Third Edition. 1897.
*Diseases of the Gall-Bladder and Bile-Ducts.* By A. W. MAYO ROBSON. 1897.
*Practical Medical Electricity.* By DAWSON TURNER, B.A., M.D. Second Edition. 1897.

From JOHN BALE, SONS & DANIELSSON, LTD., London:

*The Journal of Balneology and Climatology.* July, 1897.
*Spinal Curvatures.* By Dr. PERCY G. LEWIS. 1897.

From FRIEDR. BAYER & CO., Elberfeld:

*Clinical Excerpts.* June—August, 1897.

From CARL BELLMANN, Prague:

*Ueber die Verwendbarkeit des Gottlieb'schen Tannalbin als Darmadstringens in der Kinderpraxis.* Von MUDr. JOHANN CZEMETSCHKA. 1897. [Reprint.]

From BENNETT BROTHERS, LTD., Bristol:

*1896. City and County of Bristol. Annual Report of the Medical Officer of Health.* 1897.

From BURGOYNE, BURBIDGES & CO., London:

*Von Heyden's Non-Toxic Preparations.* August, 1897.

From CALIFORNIA MEDICAL COLLEGE, San Francisco:

*California Medical Journal.* June, July, 1897.

From J. & A. CHURCHILL, London:

*Convergent Strabismus.* By EDWIN HOLTHOUSE, M.A. 1897.
*Medical Diagnosis.* By SAMUEL FENWICK, M.D., and W. SOLTAU FENWICK, M.D., B.S. Eighth Edition. 1897.
*The Means by which the Temperature of the Body is maintained in Health and Disease.* By W. HALE WHITE, M.D. 1897.

From CHURCH MISSIONARY SOCIETY, London:

*Mercy and Truth.* July—September, 1897.

From C. J. CLAY AND SONS, London:

*Practical Morbid Anatomy.* By H. D. ROLLESTON, M.A., M.D., and A. A. KANTHACK, M.D. 1894.
*The Vertebrate Skeleton.* By SIDNEY H. REYNOLDS, M.A. 1897.

From WILLIAM F. CLAY, Edinburgh:

*Diseases of the Spinal Cord.* By BYROM BRAMWELL, M.D. Third Edition. 1895.

From THE COMMERCIAL GAZETTE JOB PRINT, Cincinnati:

*Thirty-Sixth Annual Report of the Cincinnati Hospital.* 1897.

From THE F. A. DAVIS COMPANY, Philadelphia:

*Eye-Strain in Health and Disease.* By AMBROSE L. RANNEY, A.M., M.D. 1897.

From O. H. DREYER, St. Louis:

*Medical Review.* June 5, 1897.

From EDUARDO DUBLAN, Mexico:

*Boletin del Consejo superior de Salubridad.* Tom. II. Núm. 9, 10. 1897.

From PAUL DUPONT, Paris:

*Sur un Cas très grave de Dermatite consécutive à deux Applications de Rayons X.—Pathogénie et Traitement.* Note de M. le Dr. G. APOSTOLI. 1897.

*Sur les Applications nouvelles du Courant ondulatoire en Thérapeutique électrique.* Note de M. le Dr. G. APOSTOLI. 1897.

*Sur l' Action thérapeutique générale des Courants alternatifs de haute Fréquence.* Note par MM. G. APOSTOLI et BERLIOZ. 1897.

From THE GUTENBERG PRESS, LTD., London:

*Science Siftings.* July 31, 1897.

From Dr. J. O. HIRSCHFELDER, San Francisco:

*The Cure of Tuberculosis by Oxytuberculine, with Experiments on Patients, Animals, and Cultures.* By J. O. HIRSCHFELDER, M.D. 1897.

From INGRAM BROTHERS, London:

*English Illustrated Magazine.* July—September, 1897.

From JUDD & DETWEILER, Washington:

*Catalogue of the Columbian University.* 1897.

From S. KARGER, Berlin:

*Die Anatomie und Behandlung der Geburtsstörungen nach Antefixirung des Uterus.* Von Dr. W. RÜHL. 1897.

From KNOLL & Co., Ludwigshafen, A/Rh.:

*Weitere Erfahrungen über die therapeutische Verwerthbarkeit des Tannalbin.* Von Dr. CONRAD STEIN. 1897. [Reprint.]
*Ueber das Ichthalbin (Ichthyoleiweiss), ein geschmack- und geruchloses Ichthyolpräparat.* Vorläufige Mittheilung von Dr. med. et phil. ARNOLD SACK. 1897.
*L' Ichthalbine (Albuminate d' Ichthyol), une préparation nouvelle d' ichthyol sans goût ni odeur.* Extrait d' une communication préalable par Mr. le Dr. A. SACK. 1897. [Reprint.]
*La Tannalbine contre la diarrhée des enfants et des adultes.* [1896–1897.] [Reprints.]
*Ueber die Verwendbarkeit des Tannalbin als Darmadstringens in der Kinderpraxis.* Von Dr. J. CZEMETSCHKA. 1897. [Reprint.]
*Tannalbin in der Kinderpraxis.* Von Dr. HANS OSK. 1897. [Reprint.]

From H. K. LEWIS, London:

*Notes on Malaria in connection with Meteorological Conditions at Sierra Leone.* By Surgeon-Major E. M. WILSON, C.M.G. 1897.
*Practical Muscle Testing.* By W. S. HEDLEY, M.D. 1897.

From JAMES MACLEHOSE & SONS, Glasgow:

*Human Anatomy.* By JOHN CLELAND, M.D., LL.D., D.Sc., F.R.S., and JOHN YULE MACKAY, M.D., C.M. 1896.

From MACMILLAN AND CO., LIMITED, London:

*The Action of Medicines.* By T. LAUDER BRUNTON, M.D., D.Sc., LL.D., F.R.S. 1897.
*A System of Medicine.* By Many Writers. Edited by THOMAS CLIFFORD ALLBUTT, M.A., M.D., LL.D., F.R.S. Volume III. 1897.

From JOHN MORGAN & CO., Glasgow:

*A Plea for a State Medical Service.* By JAMES ERSKINE, M.A., M.B. 1897.

From THE NEW YORK POST-GRADUATE MEDICAL SCHOOL AND HOSPITAL, New York:

*Sixteenth Annual Announcement of the New York Post-Graduate Medical School and Hospital for* 1897–98.

From OFICINA TIP. DE LA SECRETARIA DE FOMENTO, Mexico:

*Revista Médica.* Tom. X. Núm. 1—3. 1897.

From YOUNG J. PENTLAND, London

*Manual of Bacteriology.* By ROBERT MUIR, M.A., M.D., and JAMES RITCHIE, M.A., M.D., B.Sc. 1897.

From THE REBMAN PUBLISHING COMPANY, LIMITED, London:

*Archives of Skiagraphy.* No. 4. Vol. I. April, 1897.

*Diseases of Women.* By J. BLAND SUTTON and ARTHUR E. GILES, M.D., B.Sc. 1897.

*Archives of the Roentgen Ray* (formerly *Archives of Skiagraphy).* Vol. II. No. 1. July, 1897.

From HENRY RENSHAW, London:

*The Laboratory Text-Book of Public Health.* By WILLIAM R. SMITH, M.D., D.Sc., F.R.S.E. 1896.

From JOHN MORGAN RICHARDS, London:

*Medical Reprints.* June—August, 1897.

From WILLIAM ROSE & CO., Manchester:

*A History of the Fire Service and its Organizations.* [N.D.]

From THE SCIENTIFIC PRESS, LIMITED, London:

*Elementary Physiology for Nurses.* By C. F. MARSHALL, M.D., B.Sc. 1897.
*The Midwives' Pocket-book.* By HONNOR MORTEN. 1897.

From WALTER SCOTT, LTD., London:

*Hallucinations and Illusions.* By EDMUND PARISH. 1897.
*The Psychology of the Emotions.* By TH. RIBOT. 1897.

From STEENSKE BOGTRYKKERI, Christiania:

*Om Endokardit.* Af FRANCIS HARBITZ. 1897.

From GEORGE TIEMANN & CO., New York:

*Tiemann's Reprints.* No. 17. [N.D.]

From JOHN WRIGHT & CO., Bristol:

*The Pocket Therapist.* By THOS. STRETCH DOWSE, M.D. 1897.

*Diet Chart and Alimentary Index.* Suggested by W. J. KENNY. 1897.

*Our Baby.* By Mrs. LANGTON HEWER. Fifth Edition. 1897.

